# Small Insertions and Deletions Drive Genomic Plasticity during Adaptive Evolution of Yersinia pestis

**DOI:** 10.1128/spectrum.02242-21

**Published:** 2022-04-19

**Authors:** Yarong Wu, Tongyu Hao, Xiuwei Qian, Xianglilan Zhang, Yajun Song, Ruifu Yang, Yujun Cui

**Affiliations:** a State Key Laboratory of Pathogen and Biosecurity, Beijing Institute of Microbiology and Epidemiology, Beijing, China; b School of Basic Medical Sciences, Anhui Medical University, Hefei, China; University of Pittsburgh School of Medicine

**Keywords:** *Yersinia pestis*, small insertions and deletions, indel, population genomics, evolution, adaptation

## Abstract

The life cycle of Yersinia pestis has changed a lot to adapt to flea-borne transmission since it evolved from an enteric pathogen, Yersinia pseudotuberculosis. Small insertions and deletions (indels), especially frameshift mutations, can have major effects on phenotypes and contribute to virulence and host adaptation through gene disruption and inactivation. Here, we analyzed 365 Y. pestis genomes and identified 2,092 genome-wide indels on the core genome. As recently reported in Mycobacterium tuberculosis, we also detected “indel pockets” in Y. pestis, with average complexity scores declining around indel positions, which we speculate might also exist in other prokaryotes. Phylogenic analysis showed that indel-based phylogenic tree could basically reflect the phylogenetic relationships of major phylogroups in Y. pestis, except some inconsistency around the Big Bang polytomy. We observed 83 indels arising in the trunk of the phylogeny, which played a role in accumulation of pseudogenes related to key metabolism and putatively pathogenicity. We also discovered 32 homoplasies at the level of phylogroups and 7 frameshift scars (i.e., disrupted reading frame being rescued by a second frameshift). Additionally, our analysis showed evidence of parallel evolution at the level of genes, with *sspA*, *rpoS*, *rnd*, and YPO0624, having enriched mutations in Brazilian isolates, which might be advantageous for Y. pestis to cope with fluctuating environments. The diversified selection signals observed here demonstrates that indels are important contributors to the adaptive evolution of Y. pestis. Meanwhile, we provide potential targets for further exploration, as some genes/pseudogenes with indels we focus on remain uncharacterized.

**IMPORTANCE** Yersinia pestis, the causative agent of plague, is a highly pathogenic clone of Yersinia pseudotuberculosis. Previous genome-wide SNP analysis provided few adaptive signatures during its evolution. Here by investigating 365 public genomes of Y. pestis, we give a comprehensive overview of general features of genome-wide indels on the core genome and their roles in Y. pestis evolution. Detection of “indel pockets,” with average complexity scores declining around indel positions, in both Mycobacterium tuberculosis and Y. pestis, gives us a clue that this phenomenon might appear in other bacterial genomes. Importantly, the identification of four different forms of selection signals in indels would improve our understanding on adaptive evolution of Y. pestis, and provide targets for further physiological mechanism researches of this pathogen. As evolutionary research based on genome-wide indels is still rare in bacteria, our study would be a helpful reference in deciphering the role of indels in other species.

## INTRODUCTION

Currently, most of bacterial population genomic studies are based on variation of single-nucleotide polymorphisms (SNPs). However, for species with low levels of genetic diversity, SNPs can only partially shed light on the patterns of adaptive evolution. For example, Yersinia pestis, the causative agent of plague, is a highly pathogenic clone of the enteric pathogen Yersinia pseudotuberculosis. It evolved to a flea-borne pathogen ~7,000 years ago, along with an increase in severity of induced disease (1, 2). Though it has an evolutionary history for thousands of years, Y. pestis displays relatively low levels of genetic variation (3) and shows a limited number of adaptive signals; e.g., according to the genome-wide SNPs analysis of 133 Y. pestis global isolate*s*, only two genes contained positive selection signs of both an excess of nonsynonymous SNPs and homoplasies, and 20 others displayed just one of these features (4).

Compared with SNPs, small insertions and deletions (indels) are more prone to have a major influence on gene functions and corresponding phenotypes through introducing frameshifts or variable number of tandem repeats (VNTRs) (5–7). A recent analysis, scanning indels in 5,977 publicly available M. tuberculosis genomes, revealed the phenomenon of “indel pockets” (8). It showed that the sequence complexity scores around orphan indels, which had a distance of at least 100 bp with other indels, declined in regions from 7–10 bases upstream to 15–18 bases downstream of the indel position, seeming to form a pocket in the indel site. Notably, if a frameshift coding indel changes the protein sequence and causes potential deleterious effects, there will be a high selection pressure to introduce a subsequent frameshift indel to correct the reading frame (8, 9). Such sequential frameshift indels were named scars in the study of M. tuberculosis. A total of 74 unique scars were detected in 5,977 clinical isolates. Though the majority of frameshift scars were the result of direct descents from common ancestors, 6 scars suggested convergent evolution. In addition, a 1-bp homoplastic indel in *espK* was reported in an outbreak of M. tuberculosis, which is likely associated with M. tuberculosis transmissibility (6). Could the formation of “indel pockets” and selection signatures of indels, such as scars and homoplastic sites, which might be of benefit for bacterial evolution, be prevalent in other species besides M. tuberculosis?

Here we used 365 public genomes of Y. pestis with raw reads available, which had a good representation of global genetic diversity, to detect genome-wide indels in this plague bacterium. We investigated the distribution profiles of indels, and their association with sequence complexity. Meanwhile, we also screened indels that might indicate strong signals of selection and be meaningful to the evolution of Y. pestis, such as indels arising in the trunk of the phylogenic tree, sequential frameshift indels, homoplasies at the level of phylogroups, and clustered indels at the level of genes.

## RESULTS

### General features of indels in Y. pestis.

In comparison with SNPs, for a given genomic site, indels are more prone to lead to multiple types of variants. For example, indels induced by tandem repeats (TRs) could have various copy numbers in different individual genomes, which could be either insertions or deletions. In order to facilitate statistics and to discriminate between the specific genomic site and their variants, in current study we would use indel to refer to the former, and indel-variants (including deletion-variants and insertion-variants) for the latter.

A total of 2,092 small indels (≤30 bp) relative to the reference of CO92 were identified on the core genome of Y. pestis shared by ≥90% sequences of 365 globally isolated strains, including 2,032 indels on the chromosome, 33 indels on pCD1 plasmid, and 27 indels on pMT1 plasmid (Table S1 and S2 in the supplemental material). Focusing on 2,032 indels on the chromosome, we found that 58.66% of indels (*n* = 1,192) were in protein-coding sequences, 37.60% (*n* = 764) in intergenic regions, and 3.74% (*n* = 76) in pseudogenes annotated in CO92 genome (Table 1). The indel density in intergenic regions, with an occurrence rate of 1.1 indels per 1 kb, was three times as high as that in genic regions (including pseudogenes), which was 0.36 indel per 1 kb. Notably, TRs, including homo-polymeric regions, influenced the occurrence of indels. TRs accounted for 52.41% (*n* = 1,065/2,032) of total indels. Its abundance was higher in intergenic regions, with 61.13% (*n* = 467/764) indels falling in TRs, compared to a proportion of 39.43% (*n* = 500/1,268) in genic regions (including pseudogenes). TRs in intergenic regions had a density of 93 bp in every 1 kb, compared to 33 bp per 1 kb in genic regions. After excluding indels in TRs from consideration, the indel density in intergenic regions decreased, but was still twice the density in genic regions, with a rate of 0.42 indel per 1 kb and 0.21 indel per 1 kb, respectively. Besides, to our interest, for indel-variants in TRs, the ratio of insertion to deletion between intergenic and genic regions was comparable, with a ratio of 0.75 (*n* = 241/323) and 0.71 (*n* = 248/350), respectively; while for non-TR indel variants, the ratio in intergenic regions was 1.53 (*n* = 185/121), higher than that in genic regions, which was 0.79 (*n* = 344/436) (Table 1).

**TABLE 1:** Summary of detected indels on the chromosome of Y. pestis

Type	Seq len (bp)	TR			Non-TR			Total^*a*^
		ins	del	ins/del	ins	del	ins/del	
In gene	3,865,266	229	332	0.71	318	416	0.79	1,295 (1,192; 58.66%)
In pseudogene		19	18		26	20		83 (76; 3.74%)
Intergenic	788,462	241	323	0.75	185	121	1.53	870 (764; 37.60%)
Total	4,653,728	489	673	0.73	529	557	0.95	2,248 (2,032)

a If an indel site had more than one variant type, which we called indel-variants, it would be counted multiple times. Numbers in the parentheses of the “Total” column indicates the actual number of mutated positions on the chromosome and its proportion in the total 2,032 sites.

**FIG 1:**
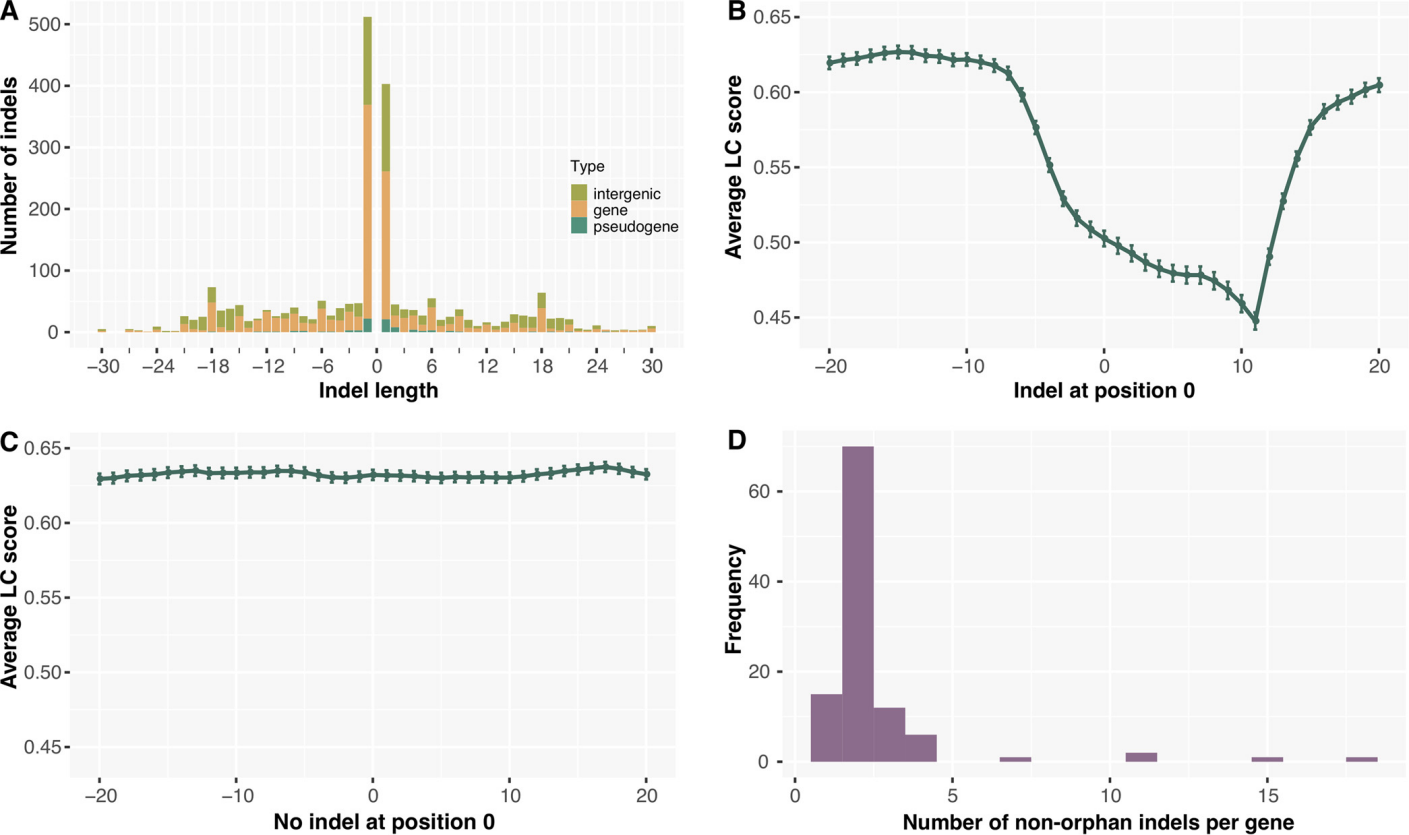
Properties of indels identified in 365 genomes of Y. pestis around the globe. (A) Indel frequency (*y* axis) is shown for different indel sizes (*x* axis) with negative and positive values indicating deletions and insertions, respectively. (B) Average linguistic complexity (LC) scores are shown for 1,479 orphan indel sites (without other indels within 100 bp) and additional 20 bases before and after the indel position (denoted by 0 on the *x* axis). The error bars denote ±1SE (standard error). (C) Average linguistic complexity (LC) scores are shown for randomly selected 1,479 non-indel sites that do not have an indel within flanking regions 100 bases upstream and downstream of the site. (D) Frequency of genes is shown as a function of the number of nonorphan indels (having adjacent indels within 100 bp) per gene.

Then we compared the frequency of in-frame indels (occurring in three or multiples of three bases) and frameshift indels. The result showed that the number of frameshift indel-variants (*n* = 1,629, 72.46%) were far more abundant than in-frame ones (*n* = 619, 27.54%), because 1-bp indels were obviously predominant in both intergenic and genic regions (Fig. 1A). But for indels >1 bp, we could see that the frequency of in-frame indels in genic regions was slightly higher than that of frameshift indels in each 3-bp window.

### Low genomic complexity contributes to the incidence of indels.

In Y. pestis, the linguistic complexity (LC) score of 2,032 identified indel positions on the chromosome, with a mean of 0.51, was significantly lower than that of the same amount of randomly selected nonindel sites, which had an average score of 0.63 (Welch’s *t* test, *P* value <2.2e-16). Similarly, the difference of Shannon’s entropy (H) score between indel and non-indel sites was also notable, with a mean of 0.93 and 0.96, respectively (Welch’s *t* test, *P* value < 2.2e-16). Our discovery was consistent with previous reports that indels were associated with low sequence complexity (8, 10, 11).

According to the definition of orphan indels (at least 100 bp away from other indels) in M. tuberculosis (8), we obtained 1,479 such sites in our data set. As referred in M. tuberculosis, our results also supported the formation of “indel pockets” in orphan indels of Y. pestis, with LC and H scores showing a decrease from 7–9 bases before the indel position to 18–19 bases after the indel position (Fig. 1B and Fig. S1A), whereas, the sequence complexity profiles around nonindel sites was generally identical (Fig. 1C and Fig. S1B). On the basis of these findings, we speculate that the phenomenon of “indel pockets” around orphan indels might also present in other prokaryotes, and more evidence in other species are needed.

### Phylogenetic contribution of indels in Y. pestis.

**FIG 2:**
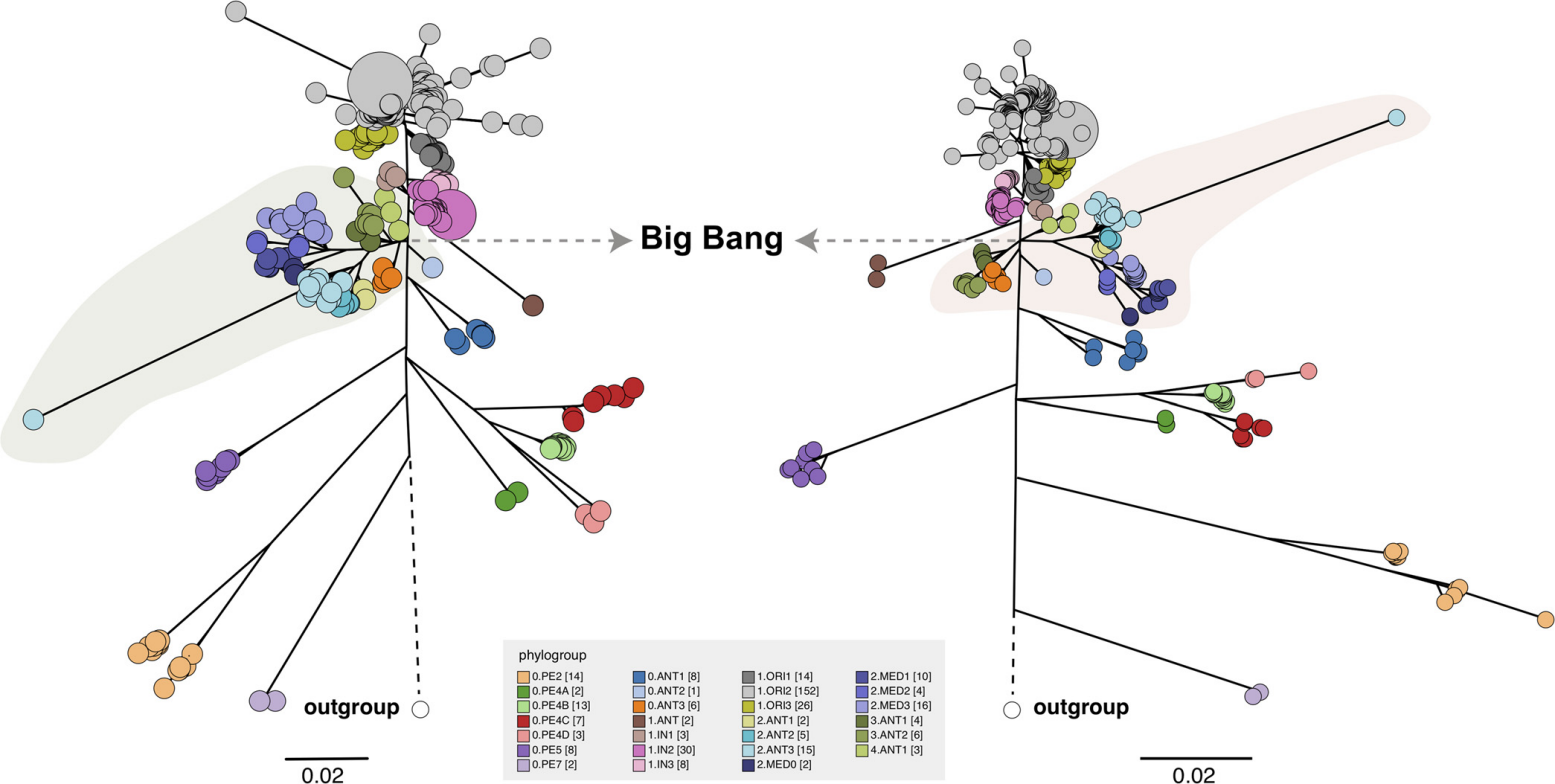
Comparation of indel-based and SNP-based phylogeny with 365 genomes of Y. pestis involved. Maximum likelihood trees, based on 2,032 indels called in ≥90% genomes (left panel) and 3,540 SNPs called in ≥95% genomes (right panel), were visualized in GrapeTree tool. Nodes are color coded by previously designated phylogroups (see legend at the bottom) (4, 38). Inconsistency between the two phylogenies around the Big Bang polytomy was highlighted with green and orange shades.

To investigate the contribution of indels to phylogenetic signal in Y. pestis, we reconstructed the phylogenetic tree of Y. pestis based on the presence–absence pattern of 2,032 indels on the chromosome (see Materials and Methods). In general, the tree topology was identical to that of standard SNP-based phylogeny, with 3,540 SNPs involved (Table S3), except that the indel-based phylogeny had a lower resolution around the Big Bang polytomy (4). The populations of 0.ANT2 and 0.ANT3 in branch 0, which should diverge prior to the node of the Big Bang, were now placed together with phylogroups in branch 2-4, although each population still could be apparently discriminated from others (Fig. 2).

### Varied forms of selection signals indicated by indels.

**FIG 3:**
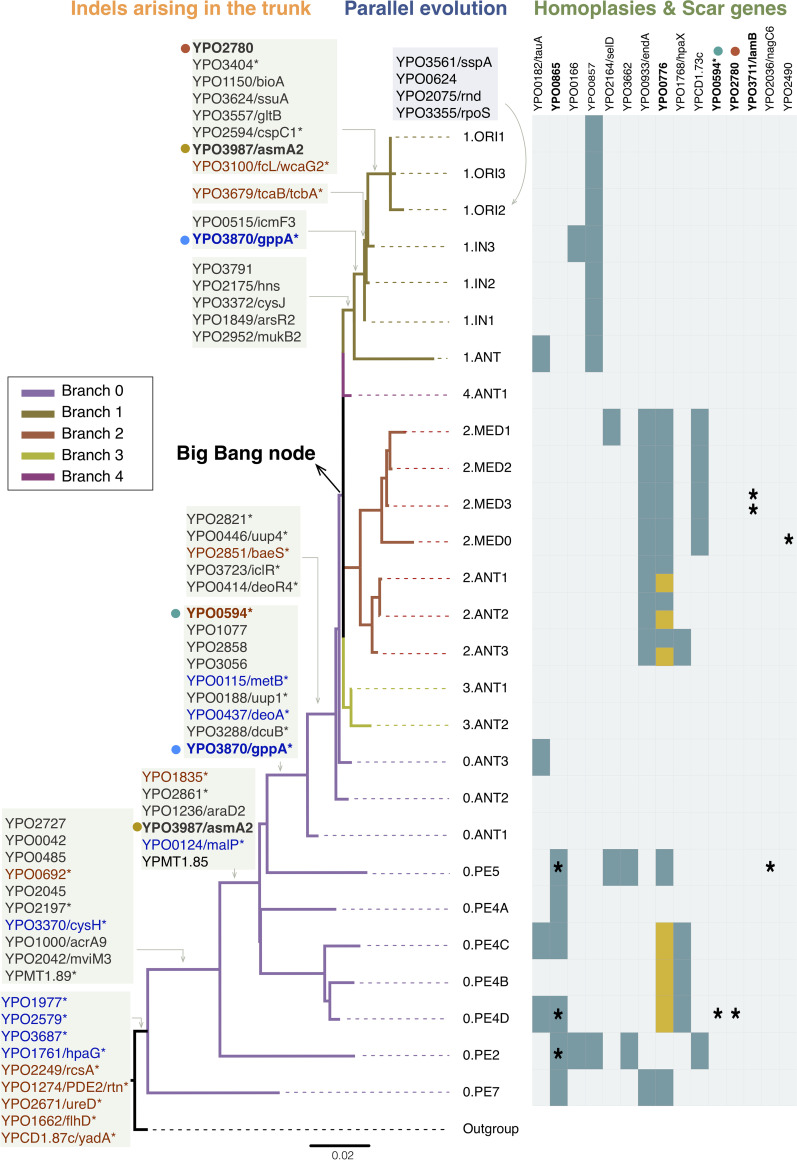
Schematic phylogeny with genes carrying different forms of selection signals. In order to support visualization, only phylogroups are displayed in the schematic phylogeny, and the branch length is proportional to that of the SNP-based phylogenetic tree. Gene IDs in bold correspond to genes showing more than one form of selection signs or fixed in the trunk more than once. For indels arising in the trunk of the phylogeny, gene IDs in blue represent pseudogenes that might be important in central/intermediary metabolism, while gene IDs in red represent pseudogenes that might be important in the pathogenesis of other *Yersinia* species. Pseudogenes are labeled with asterisk after the gene ID. Homoplasies and scar genes located in genic regions are shown in the rightmost heatmap, with corresponding phylogroups highlighted in turquoise or yellow (homoplasies) or marked by an asterisk (scar genes). Yellow filled box in gene YPO0776 represents another homoplastic site occurring in this gene.

Although low-frequency indels were abundant on the chromosome, with 68.80% indel-variants occurring in ≤5 strains (Fig. S2), we still identified 83 indels (including 12 sites with more than one indel-variants) fixed in the trunk of the phylogeny, with populations before or after a specific inner node sharing the same variation, which might be related to adaptive evolution of Y. pestis. Among the 83 indels sites, 79 indels were located on the chromosome, 3 indels on pMT1 plasmid and 1 indel on pCD1 plasmid. And 46 indels were involved in 44 protein-coding regions (including pseudogenes) (Fig. 3 and Table S4), and the other 37 indels in 35 intergenic regions (Table S5). Notably, 66 out of 83 sites (with 35 in genic regions and 31 in intergenic regions) were insertions compared to reference genome of CO92. Due to the fact that CO92 belonged to 1.ORI1, a most recently evolved phylogroup in Y. pestis, it actually revealed that deletions were far more predominant than insertions in the key genomic changes after Y. pestis diverged from its ancestor.

Disruption of the open reading frame of a gene by indels, especially frameshift indels, could considerably affect gene function. Although gene inactivation is a genome decay or is harmful to the gene itself, it could provide fitness advantages at the species level, and contribute to host adaption or virulence, such as the parallel inactivation or loss of *cadA* gene during the emergence of *Shigella* lineages (12), the inactivation of PDE2 (*rtn*), *rcsA*, *ureD*, and *flhD* in Y. pestis during initial adaptation to flea-borne transmission (13–18). Therefore, we speculate indels in genic regions, which occurred on the stem of the phylogenic tree, may have a fitness advantage and might be the result of natural selection. Focusing on the 46 indel sites in genic regions, more than three-quarters (*n* = 35) could theoretically disrupt the reading frame and lead to gene inactivation. As expected, 23 sites were associated with 22 annotated pseudogenes in either the CO92 or 91001 genome. In Y. pestis, 9 pseudogenes caused by frameshift indels were reported to be potentially important in central/intermediary metabolism (7). To our interest, 5 of them (*gppA*, *cysH*, *metB*, *deoA*, and *malP*) had 6 fixed indels in the trunk of the phylogenetic tree of Y. pestis (Fig. 3 and Table S4). The other 4 pseudogenes (*hpaG*, YPO1977, YPO2579, and YPO3687) emerged before the most recent common ancestor (MRCA) of all modern Y. pestis genomes. Moreover, there were 6 other fixed indels falling in 6 pseudogenes related to putative pathogenicity islands or virulence determinants, as well as five virulence-related pseudogenes, *yadA*, PDE2 (*rtn*), *rcsA*, *ureD*, and *flhD*, which emerged after the split from Y. pseudotuberculosis or before the MRCA all modern Y. pestis genomes (13, 15–20) (Fig. 3 and Table S4). In short, our results confirmed that frameshift indels contributed to key changeovers of metabolism and pathogenicity during evolution of Y. pestis.

Scarred genes (referred to as a second frameshift indel restoring the reading frame disrupted by one frameshift) (8) and homoplasies are also signs of selection. In the analysis of 365 global isolates of Y. pestis, we identified 7 unique scars in 6 genes (Fig. 3 and Table S6), with 3 scars having a long distance (>500 bp) between frame-shifting and restoring indels, which may alter the gene product. The occurrence of 4 scars were due to a common evolutionary ancestor, as isolates having identical frameshifts belonged to the same phylogroup, whereas the other 3 scars showed signs of convergent or parallel evolution. In YPO0865, the same frameshift scar was shared by 3 isolates from distinct phylogroups (0.PE2, 0.PE4D, and 0.PE5). The disruption of gene *lamB* by one nucleotide deletion was restored via 2 independent frameshifts, one 2-bp deletion, and one 1-bp insertion. Furthermore, more than 200 homoplasies were detected in our data set. To reduce the influence introduced by low-frequency mutations, which might be deleterious but not yet been purified, our investigation centered on homoplasies that were shared by all strains in at least two phylogroups with ≥5 strains. Finally, 32 indels were identified that independently appeared in 2 to 8 phylogroups (Table S7). Consistent with reported features of variable number of tandem repeats (VNTR) in Y. pestis (21, 22), YPO0776 showed the highest diversity among homoplastic sites, with 4 indel-variants (involved in 2 indel sites) appearing in different phylogroups (Fig. 3 and Table S7).

### Strong parallel evolution signals at the gene level emerged in isolates from the northeast of Brazil.

By screening the density of indels along the genome, we found 5 genes, *sspA*, *rpoS*, *rnd*, YPO0623, and YPO0624, with extraordinarily more indels enriched than common genes (Fig. 1D). Surprisingly, except for YPO0623, the indel-variants, as well as substitutions, in these 4 genes, mainly occurred in isolates from Northeastern Brazil (23) (Table 2). To verify the possible influence of sequencing data quality, we counted the number of indels and average sequencing depth for each sample, and compared them between 47 Brazilian samples that displayed a remarkably high number of indels in 4 genes, and the other 72 Brazilian samples without such clustered mutations. As there was no significant difference between these two data sets (Welch’s *t* test, *P* = 0.59 and 0.4, Fig. S6A and B), it indicated that the unusual high rates of indels in Brazilian samples were not caused by the potential bias lead by data quality, which was also supported by random sampling to keep the same number of strains between two groups (Fig. S6C and D).

**TABLE 2:** Statistics of variations located on genes with unusual number of indels

Gene name	Gene product	Gene len (bp)	No. of indels (Brazil/total)	No. of SNPs (Brazil/total)
YPO3561/*sspA*	Stringent starvation protein A	642	14/15	5/8
YPO0624	Hypothetical protein	1,452	14/19	4/9
YPO2075/*rnd*	Ribonuclease D	1,122	7/7	3/3
YPO3355/*rpoS*	RNA polymerase sigma factor RpoS	999	10/11	4/6
YPO0623	Aminotransferase	1,233	1/12	0/5

**FIG 4:**
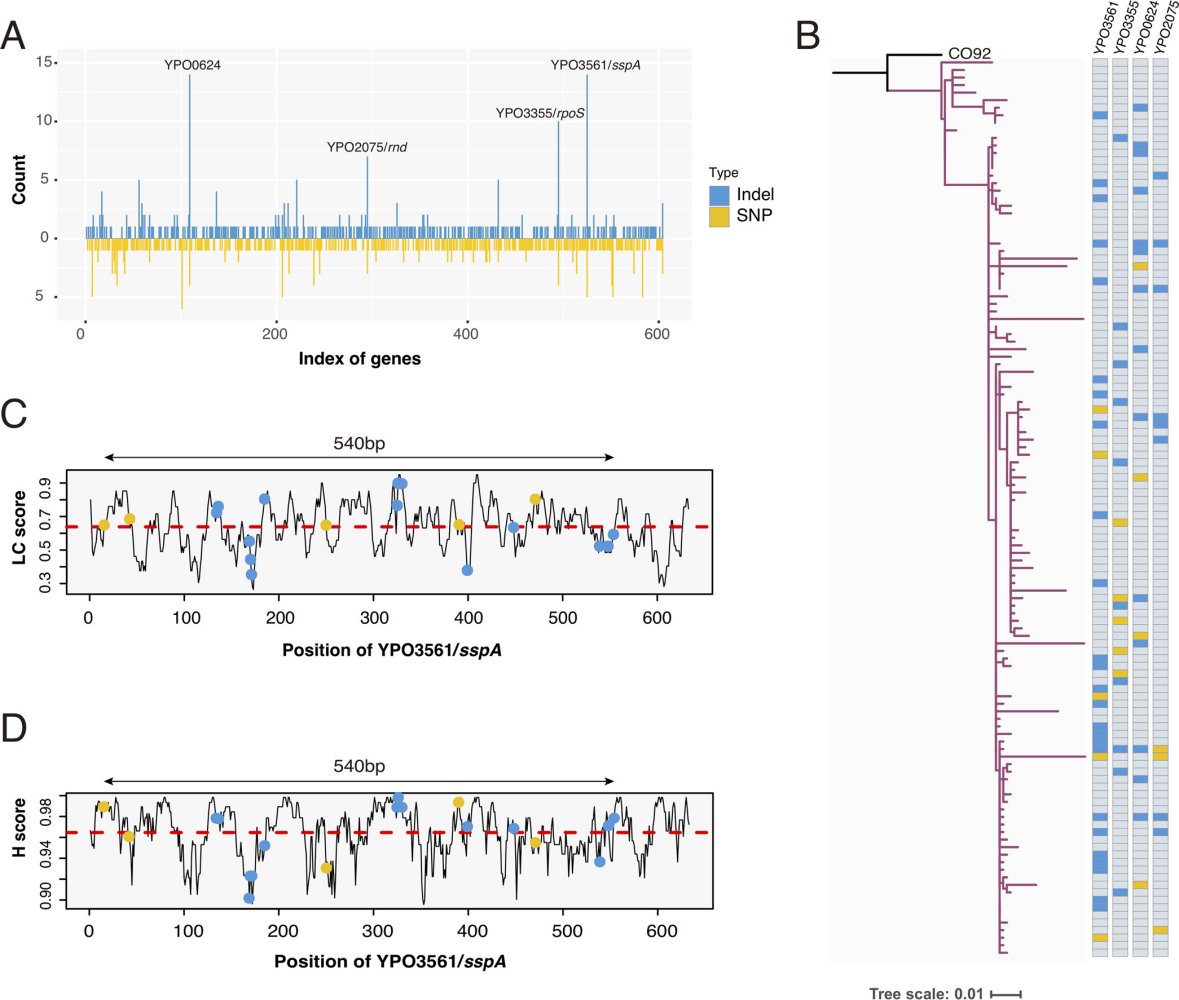
Properties of mutations identified in 119 isolates from Brazil. (A) Bar plot showing the count of SNPs and indels in 604 genes, which had either substitution variations or indels, or both occurring in Brazilian isolates. (B) Maximum likelihood tree of 119 Brazilian genomes. Isolates with mutations in four genes under strong selection pressure are marked in blue (indels) and yellow (SNPs) colors in the right heatmap. (C and D) Sequence complexity profiles for gene *sspA*, quantified by LC score and H score, respectively. Red dashed line indicates the average score of the entire gene. Indels and SNPs identified in this gene are marked with blue and green dots, respectively, along the gene length (*x* axis).

We then recalled both indels and SNPs for 119 genomes isolated from Brazil (23), which all belonged to a sublineage of 1.ORI2 (Fig. S3). A total of 502 SNPs and 532 indels were identified, with 604 genes/pseudogenes involved (Table S8). Statistics revealed that these 4 genes (*sspA*, *rpoS*, *rnd*, and YPO0624) did have an apparently higher number of variations, especially indels, than other genes (Fig. 4A and Table S9). Isolates with mutations in the 4 genes were scattered across the phylogenetic tree (Fig. 4B). However, no temporal signals were found between mutants of the 4 genes and other strains (Fig. S4).

Notably, gene *sspA*, encoding stringent starvation protein A, had most variations clustered in Brazilian isolates, including 14 indels and 5 SNPs within 540 bp regions. To investigate the impact of sequence composition on mutations, we analyzed the distribution of mutations along the sequence of *sspA*, together with genomic complexity scores. We found that both SNPs and indels were interspersed along the gene with a wide range of LC and H scores, instead of being restricted to regions with low sequence complexity (Fig. 4C and D). The same trend held true for the other 3 genes (Fig. S5).

## DISCUSSION

Indels are an important source of sequence variations and are prone to cause phenotypic effects compared with substitutions. However, most population genomic studies in bacterial genomes relied on SNPs, because accurate inference of indels from next-generation sequencing data is still challenging (24). Though some indel calling tools have been developed, their achieved results varied widely in terms of sensitivity and specificity, far lagging behind that of SNP callers (24–26). In addition, it is difficult to exclude the potential role of reference bias and the false-discovery rate of indel callers under the impact of variable data quality, including extremely short read length and relatively low sequencing depth along genomes. For example, the raw reads of 93 public Y. pestis genomes sequenced in the early stage of high throughput sequencing had a read length of just 35 bp, so it might be difficult to accurately identify indels longer than the read length. Therefore, in this study we used a strict quality filter to exclude the public samples of poor sequencing depth and to remove indels without enough supporting reads. Furthermore, according to a benchmark research, which indicated different indel callers could reliably detect indels ≤29 bp at sensitivities approximately 100% (24), we focused on indels within 30 bp in this study to ensure both sensitivity and accuracy.

Distribution analysis of indels in Y. pestis showed an abundance of frameshift indels, especially single nucleotide indels, consistent with indel properties in varied species (8, 27, 28). As a majority of one nucleotide indels appeared in only one or two genomes, it is highly possible that those indels were deleterious but not yet purified (Fig. S2B). An alternative explanation is that the dominance of 1-bp indels might follow a power-law behavior that widely exists in biological and other fields, as reported in a study of indels in the human genome, but other potential mechanisms cannot be ruled out (27). After excluding 1-bp indels, as expected, we observed a higher ratio between in-frame and frameshift changes in genic regions (Fig. 1A), which suggested a preference of low fitness cost for indels > 1 bp. Intriguingly, we found that the high incidence of indels in the intergenic had certain relationship with the skewed distribution of TRs along the genome, because the exclusion of TR-related indels causes a reduced ratio, from 3:1 to 2:1, between the indel density in the intergenic and that in genic regions. The high density of TRs in intergenic regions is likely because they are less constrained by selection, so indels in the intergenic could have a low fitness cost. In addition, it was noteworthy that in the non-TR regions, the ratio of intergenic insertion to deletion was twice higher than that in genic regions, while no such bias was found in TR-related indel-variants and their ratios were comparable to that of non-TR indel-variants in genic regions. In order to clarify the possible molecular mechanism for more frequent insertions than deletions falling in non-TRs of intergenic regions, further efforts are needed.

As shown in several other species (8, 10, 11), our findings confirmed that indels in Y. pestis were also associated with low sequence complexity, and the formation of “indel pockets” around orphan indels, which was firstly discovered and named in the analysis of clinical M. tuberculosis isolates, held true for Y. pestis as well. The findings of “indel pockets” in both M. tuberculosis *and* Y. pestis led us to propose that this phenomenon might also exist in other prokaryotes, which needs verifications in more bacterial species.

Indels, especially those disrupting the reading frame of a gene, could significantly alter protein function and result in a possibly large changes in fitness. Thus, the occurrence of indels in the trunk of the phylogenetic tree representing key changes during the evolution of Y. pestis, as well as scarred genes with sequential indels independently emerged in distinct genomes, and homoplasies fixed in evolutionarily distant phylogroups, might be under high selection pressures and provide driving force in the adaptation of Y. pestis to new hosts or fluctuating environments. As expected, we found 83 indel sites occurring on the stem of the phylogeny, and 23 out of 46 sites in genic regions were associated with annotated pseudogenes. Pseudogenization could contribute to the increase of virulence when genes are inactivated and is also a sign of genome decay, which makes the genome more streamlined and then facilitates the development of acute disease after reducing unnecessary metabolic pathways (7, 29, 30). Interestingly, we confirmed that the formation of 9 frameshifted pseudogenes, which were reported to be putatively essential in central/intermediary metabolic pathways (7), all happened in the trunk of the phylogenetic tree. Besides, some indels occurring in annotated pseudogenes were related with potential virulence determinants. Combining with the known inactivation of PDE2 (*rtn*), *rcsA*, *ureD*, and *flhD* by indels after prehistoric plague (13, 15–18) and the pseudogene *yadA* in pCD1 plasmid shared by all Y. pestis (19, 20), we conclude that indels arising on the stem could play an important role in speciation and evolution of Y. pestis. In addition, investigations of scarred genes and homoplastic indels also provide us information about the genomic plasticity of indels in adaptive evolution of Y. pestis. Based on indels, we summarized all possible genes (or pseudogenes) that were likely under selection pressures in Fig. 3. However, the functions and roles of some genes or pseudogenes remain unclear, which could be alternative targets for subsequent experimental study of Y. pestis.

An experiment of 115 Escherichia coli populations documented a strong pattern of parallel evolution at various levels (i.e., genes, operons, and functional units) in response to high temperature (31). Recently, parallel evolution at the level of genes was observed in natural populations of Y. pestis, with mutations clustered in a biofilm-associated gene, *rpoZ*, in isolates from a plague reservoir in Guertu, China (32). The *rpoZ* mutants had a significantly higher level of biofilm formation capability and their occurrence was proved to be associated with colder and drier climates. In this study, we found significantly high abundance of variations, especially indels, enriched in 4 genes, *sspA*, *rpoS*, *rnd*, and YPO0624, in strains isolated from Northeastern Brazil, which were highly similar to our previous study revealing strong selection pressures on the *rpoZ* gene (32). Among them, mutations in gene *sspA* were far more frequent. The stringent starvation protein A (SspA), encoded by *sspA*, is an RNA polymerase associated protein and plays a part in acid tolerance, virulence gene expression, and adaptation to nutrient starvation (33–35). Besides, RpoS, the RNA polymerase sigma subunit, encoded by *rpoS*, is a global regulator of general stress response and is important for bacteria survival in adverse conditions (36, 37). The roles of YPO0624 and RNase D encoding gene *rnd* in Y. pestis are yet unclear. The strikingly unexpected number of parallel mutations enriched in these four genes indicate strong signs of positive selection, which might be beneficial for the adaptation of Y. pestis to fluctuating environments, although the possible related environmental stresses and phenotypic changes are unknown. Moxon et al. proposed a hypothesis that elevated mutation rates at specific loci (contingency loci) could promote adaptive potential via random phenotypic alternations in face of unfavorable environments (5). This phenomenon was merely observed in Brazilian isolates in our data set, which might attribute to enriched sampling in the country, as genomes from Brazil accounted for 32.60% (*n* = 119/365) of total sequences. Like samples from Guertu, Brazilian strains were also collected annually over a long time period (from 1966–1984) in a small-scale ecosystem —the northeast region of Brazil—and had a low genetic diversity as they were assigned to an independent sublineage of phylogroup 1.ORI2. Therefore, we speculate that if increasing the sample size, especially intensive sampling for many years in a confined area, similar dense mutations occurring within other populations would be captured, which can be helpful to interpret further mechanism of adaptive evolution in Y. pestis.

In conclusion, the genome-wide indel analysis of 365 Y. pestis genomes enriches our knowledge about general features and distribution characteristics of indels in Y. pestis and provides insights into varied forms of selection signals shaped by indels. We note that “indel pockets” in orphan indels also exist in Y. pestis, and in combination with report in M. tuberculosis (8), we speculate this phenomenon could perhaps exist in other prokaryotes. Phylogenetic analysis revealed that indel-based phylogeny could basically reconstruct the phylogenic relationship of major phylogroups in Y. pestis, compared with the topology of SNP-based phylogenic tree, except a lower resolution around the Big Bang node. Meanwhile, we detect a total of 83 indels sites fixed in the trunk of the phylogeny, 7 unique scars, 32 homoplasies at the level of phylogroups, and 4 parallel evolution signs at the level of genes, which might be fitness advantage under certain selection pressures and contribute to the adaptive evolution of Y. pestis. Some genes (or pseudogene) with such indels located, are poorly characterized and could be alternative targets for further study. Together, our study could offer the basis for further exploration of indels in the adaptive plasticity of Y. pestis genome and other species.

## MATERIALS AND METHODS

### Data set preparation.

A total of 365 representative modern genomes and 12 ancient genomes from prehistoric plague with short reads publicly available were used in this study (Table S1). We referred EnteroBase (38) for the selection of representative modern genomes. When it was published, it included 1,368 modern and ancient genomes of Y. pestis available in October 2019. Because several hundreds of genomes isolated from Brazil (23) and Madagascar (39) over short time periods showed poor levels of genetic variation (having 0 core genome multilocus sequence typing allelic difference, called HC0 group), then Zhou et al. randomly selected one strain from each HC0 group to reduce sample redundancy, which resulted in a total of 622 modern genomes and 56 ancient genomes (http://enterobase.warwick.ac.uk/a/21975). In order to avoid indels called by wrong assemblies in poor sequencing regions and to verify the reliability of indels, we only considered genomes with raw data available in this work. Among the 622 modern genomes, only 378 strains had raw sequencing reads available. As these 378 genomes had a good representation of known genetic diversity of Y. pestis (except for lacking genomes in Branch 4, with only one genome of phylogroup 4.ANT1 involved), we chose them as the data set of current study, and included two additional genomes assigned to phylogroup 4.ANT1. The raw reads were downloaded from NCBI SRA database and trimmed by Trimmomatic (v0.38) software (40) with a minimum read length of 20 bp and quality score of 20. We reassessed the data quality after trimming and excluded 15 isolates with less than 20X sequencing depth and covering less than 85% of the CO92 genome (Accession No. GCF_000009065.1) length. Then a final data set of 365 modern genomes was used in this study, including 119 genomes isolated from Brazil (23). The data quality statistics are included in table S1.

### Genome assembly and SNP calling.

The 365 modern genomes were assembled *de novo* using SPAdes genome assembler (v3.13.0) (41). Then NASP software (v1.1.2) (42) was used to call SNPs for modern genomes based on both *de novo* assemblies and their short reads, with the CO92 genome as reference. All 365 *de novo* genome assemblies were aligned against reference using MUMmer (v3.23) (43). For short reads, BWA MEM (v0.7.17) aligner (44) and UnifiedGenotyper of the Genome Analysis Toolkit (GATK, v3.8) (45) were used to identify SNPs supported by at least 10 reads and 90% allele frequency. SNPs located in repetitive regions, detected by blastn (v2.8.1+) with a minimum sequence identity of 95%, and Tandem Repeats Finder (v4.07b) (46) with a minimum alignment score of 50, were excluded for further analysis. Only SNP sites shared by at least 95% genomes and derived by both *de novo* assemblies and short reads were finally retained. Y. pseudotuberculosis IP32953 was a commonly used outgroup for Y. pestis; thus, for a given SNP site, a 201-bp sequence with 100 bp upstream and downstream the position was extracted and blasted against the chromosome genome of IP32953 (accession no. AE017042.1), to get its corresponding allele. Finally, a total of 3,540 SNPs were identified within 365 modern genomes (Table S3). The above method was also applied to identify SNPs in 119 Brazilian isolates without mapping to IP32953.

### Indel calling.

Before indel calling, the GATK IndelRealigner module was used to perform local alignment of reads around gapped regions. Then aligned bam files were analyzed using GATK HaplotypeCaller with “-stand_call_conf 100 -ploidy 1 -ERC BP_RESOLUTION.” The output gvcf files for each isolate were merged by GATK GenotypeGVCFs. Then, the combined vcf file was further processed to retrieve indels that were supported by at least 10 reads and 85% frequency. We focused on indels ≤30 bp in this study. And indel sites having more than 4 variation types or being shared by less than 90% genomes were excluded from further analysis.

### Classification of indels associated with tandem repeats.

Tandem Repeats Finder was used again to identify all possible homo-polymeric sequences and other tandem-repetitive regions in CO92 reference genome. In order to report all repeats, even those with a relatively short length, we set the minimum alignment score to 1. For a given indel site, located in the identified tandem-repetitive regions, if it belonged to a homopolymer or was similar to the identified motif of tandem repeats, then it was called a TRF indel. For sites not located in tandem-repetitive regions in the reference genome, if one indel variant could be a motif for other indel variants or adjacent sequences in the reference genome, then it was also referred to as a TRF indel.

### Phylogenetic analysis.

Firstly, we generated a multiple sequence alignment by concatenating all SNP sites. Then a maximum likelihood tree was reconstructed by using IQ-TREE (v1.6.6) (47) with GTR+G model and 1,000 fast bootstrap replicates. Next, as we excluded indels with more than four variation statuses, we used A, G, C, T to represent each indel-variant, and gaps for unsure or undetected sites in an isolate. A new indel matrix, similar to the SNP matrix, was generated. Then, all indel sites were concatenated to build a maximum likelihood tree by using IQ-TREE with JC model and 1,000 fast bootstrap replicates. Finally, the SNP-based phylogeny and indel-based phylogeny were visualized in GrapeTree software (v1.5.0) (48), respectively.

### Identification of indels occurring in the trunk of the phylogeny.

For a given indel site, we counted the number of mutants in each phylogroup. Then, we focused on sites that were present in all genomes of a specific phylogroup. We allowed a maximum gap tolerance of 20% for each phylogroup, because some sites might be treated as gap after strict screening criteria in indel identification. Taking their orders that appeared in the phylogeny into consideration, we manually judged whether the indels were fixed in the trunk of the phylogeny or not. For 0.PE7, as it was the most basal modern clade containing only two genomes, sites that existed in at least one genome were considered. Then, we manually checked their variation status in two 0.PE7 genomes and 12 ancient genomes from prehistoric plague, with a lower depth requirement (>=3X), to exclude sites that were specific to 0.PE7 genomes, and finally obtained mutations that were shared by strains located in the most basal clades.

### Measurement of genomic complexity.

The FindingInfo tool (v1.0.0) (8) was used to quantify the sequence complexity for each position in the CO92 reference genome with a window size of 21 bp, including 10 bases upstream and downstream of the site. FindingInfo considers two different methods, linguistic complexity and Shannon’s entropy, to measure the sequence composition (8, 49, 50). The linguistic complexity (LC) score is calculated based on the nonrepetitive combinations of DNA nucleotides in different lengths along the sequence with their orders considered, while the Shannon’s entropy (H) score only focuses on the frequencies of four bases in the genome without considering their orders. The LC scores and H scores for specific regions were then extracted from the output files.

### Identification of homoplasies and scarred genes.

We used homoplasyFinder (v2020-08-05) (51) to identify homoplastic indels, on the basis of the indel matrix, with A, G, C, T representing indel-variants in each site, and the SNP-based phylogeny. Then isolates with mutations in each site were assigned to phylogroups. Only homoplastic sites, shared by all genomes in at least two phylogroups with ≥5 strains, were included for following analysis. And these phylogroups were required not to be clonal populations (i.e., direct descendants inherited from a common ancestor). For example, if an indel occurred in all strains from 2.MED1, 2.MED2, and 2.MED3 phylogroups, which formed a specific sublineage, it would be ignored, but if it was shared by strains in 2.ANT3 and 2.MED3, then it would be treated as a homoplastic site. In addition, sequential frameshift indels were selected using local python script according to the definition of scarred genes (8).

### Statistical tests.

The statistical tests were conducted in R Statistical Software (v3.6.1). We used the functions var.test() and t.test() to compare variances and means of two distributions. For samples having unequal variances, we performed Welch’s *t* test with “var.equal = FALSE” argument in function t.test().

### Data availability.

Publicly available genomes from NCBI SRA database are listed in supplemental Table S1.
