# Supplementary material for: Small Insertions and Deletions Drive Genomic Plasticity during Adaptive Evolution of Yersinia pestis
Source: Microbiol Spectr. 2022 Apr 19;10(3):e02242-21. doi: 10.1128/spectrum.02242-21 (PMC9248902; doi:10.1128/spectrum.02242-21)
Supplement: SUPPLEMENTAL FILE 1 — Supplemental material. Download spectrum.02242-21-s001.pdf, PDF file, 1.7 MB [file spectrum.02242-21-s001.pdf]

Supplementary Information for "Small insertions and deletions drive genomic plasticity during adaptive evolution of *Yersinia pestis*"

by Yarong Wu, Tongyu Hao, Xiuwei Qian, Xianglilan Zhang, Yajun Song, Ruifu Yang and Yujun Cui

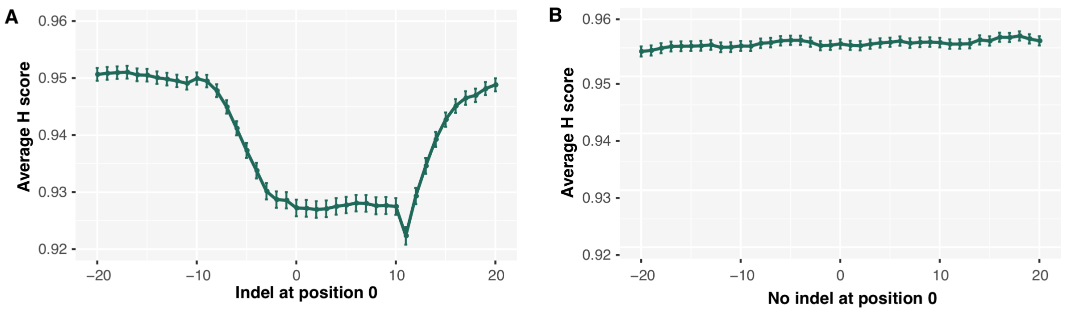

FIG. S1. (A) Average Shannon's entropy (H) scores are shown for orphan indel sites (n=1,479, without other indels within 100bp) and additional 20 bases before and after the indel position (denoted by 0 on the x-axis). The error bars denote ±1SE (standard error). (B) Average Shannon's entropy (H) scores are shown for randomly selected non-indel sites (n=1,479) that do not have an indel for 100 positions upstream and downstream of the site.

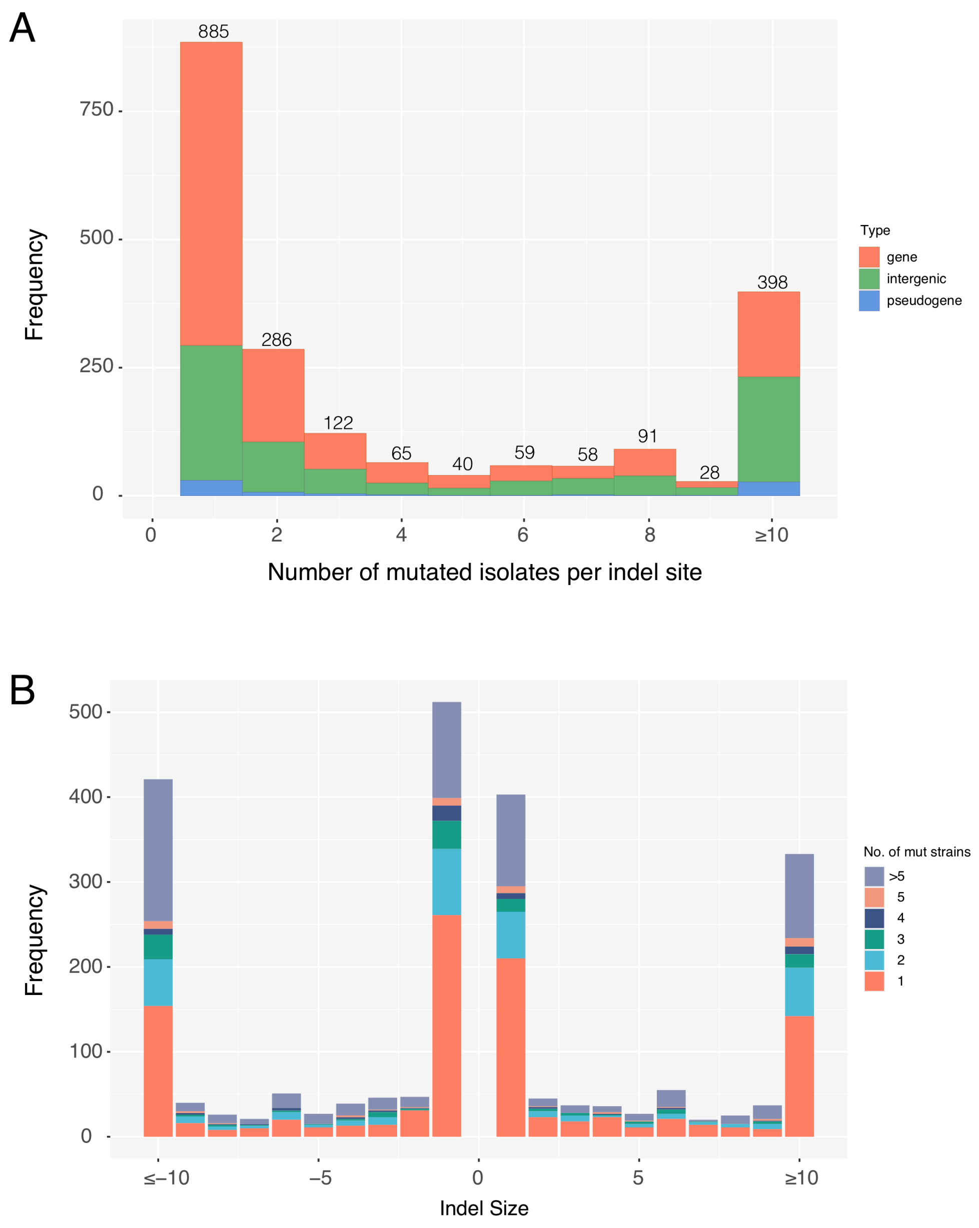

FIG. S2. Statistics about the frequency of mutated isolates per indel site and their distribution along different indel sizes. (A) Histogram showing the distribution of mutated isolates per indel site. (B) Histogram showing the distribution of mutated isolates in different indel sizes.

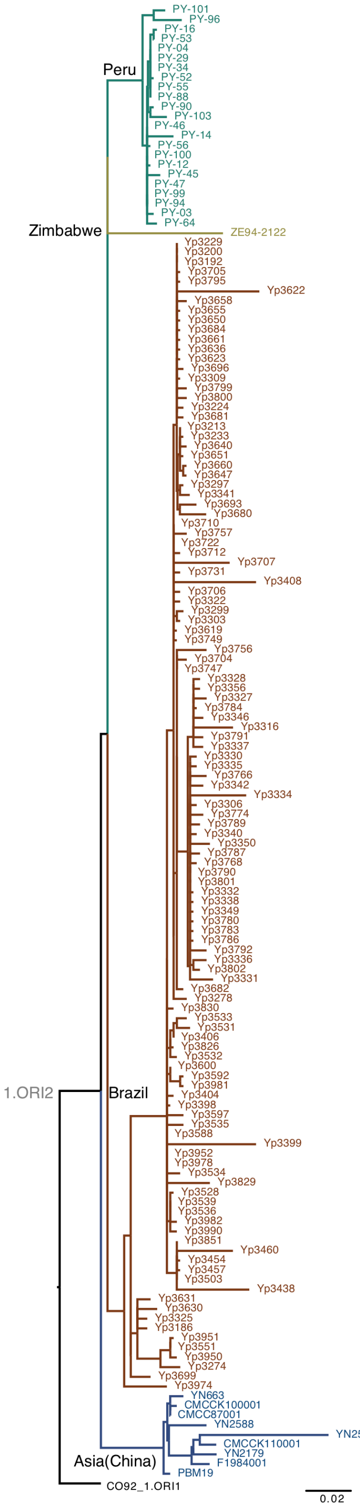

FIG. S3. Maximum likelihood tree of 152 isolates in 1.ORI2 phylogroup. The tree was reconstructed on the basis of 654 SNPs, which were identified in core genomes existing in >=95% genomes. Colored sub-lineages indicate different isolation sources, which were labeled in black text at the node of each sub-lineage.

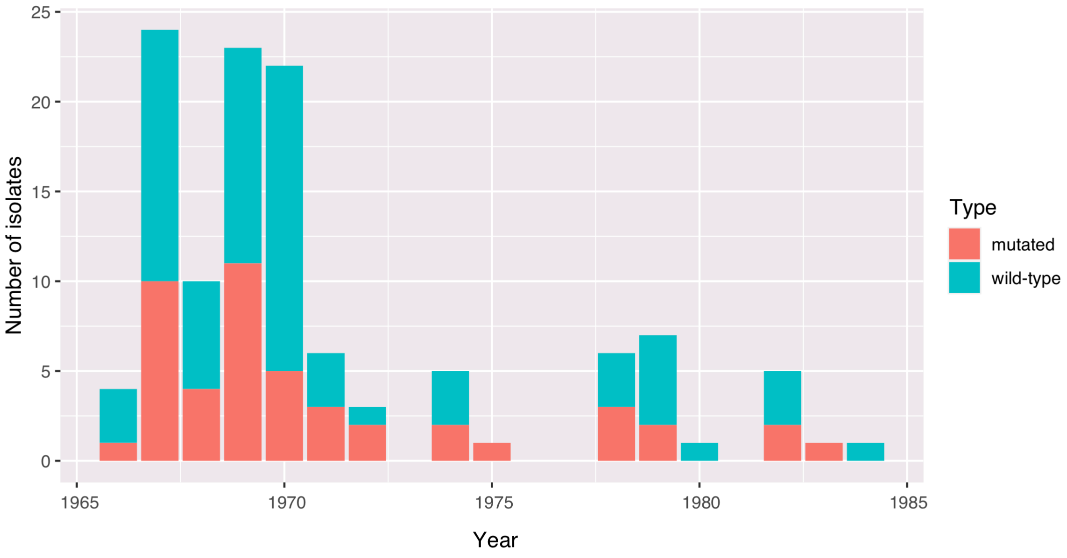

FIG. S4. Bar plot of the sampling distribution over year for 119 isolates from Brail. Strains with either SNP or indel mutations in genes *sspA*, *rpoS*, *rnd*, and YPO0624 were labeled as 'mutated', while others as 'wild-type'.

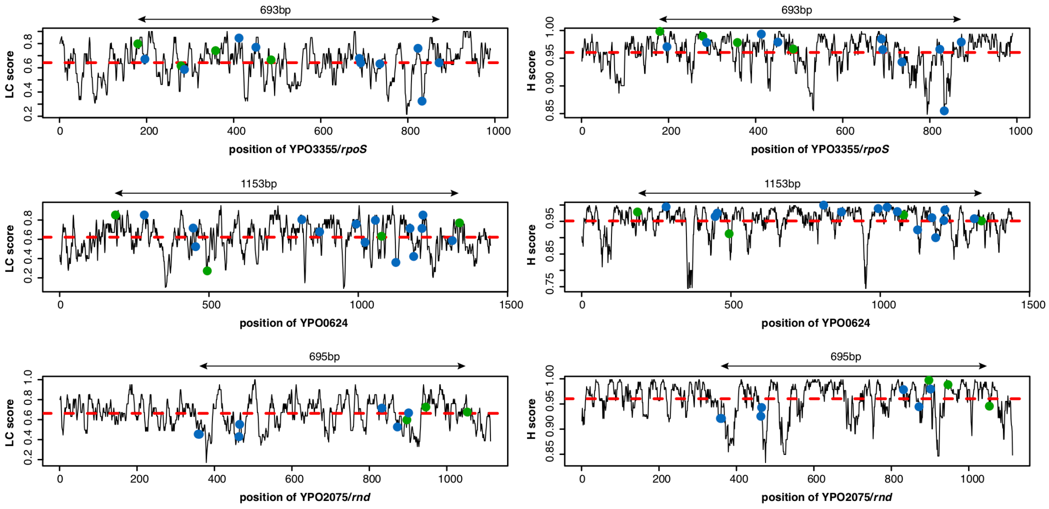

FIG. S5. Sequence complexity profiles for gene *rpoS*, YPO0624 and *rnd*. Sequence complexity (LC score: linguistic complexity score, H score: Shannon's entropy (H) score) for these 3 genes is shown along the gene length (x-axis), with red dashed line indicating the average score of the entire gene. Indels and SNPs identified within the gene are marked with blue and green dots, respectively.

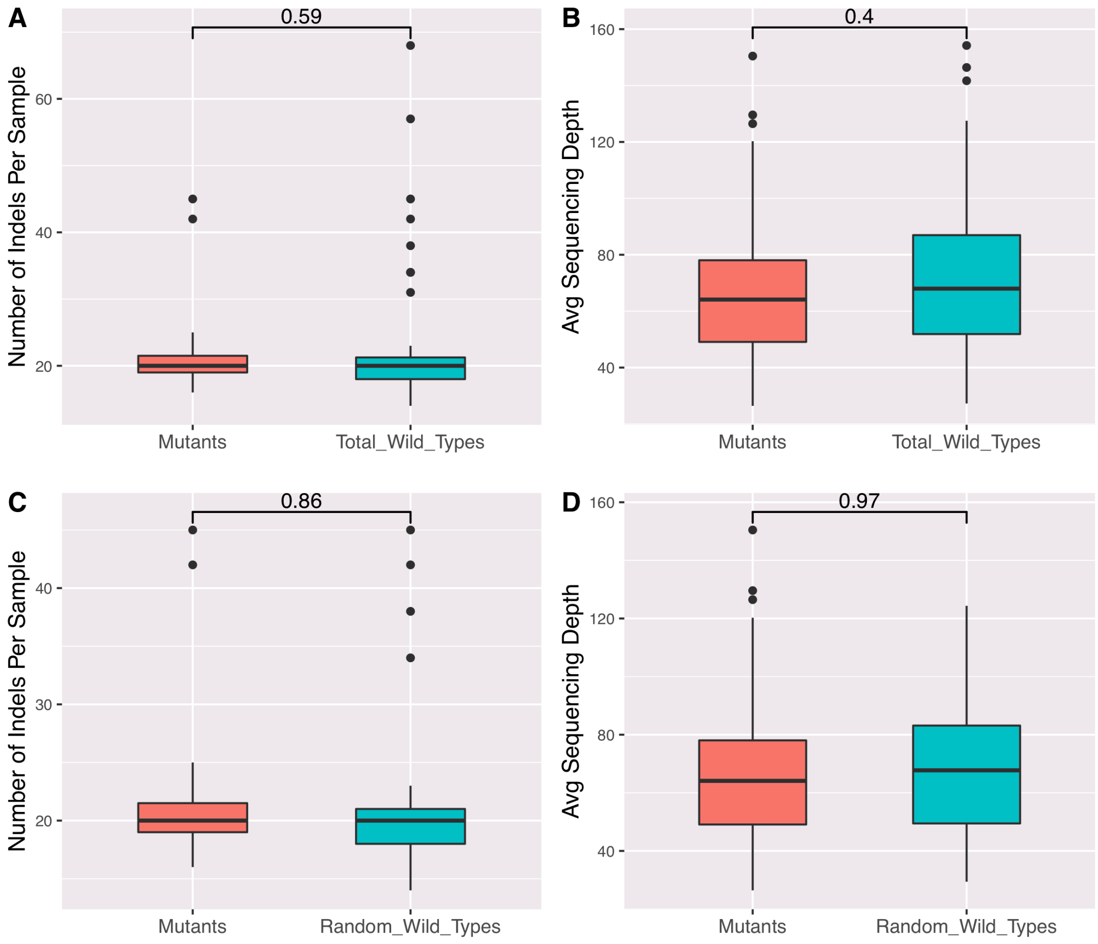

FIG. S6. Comparison between Brazilian samples that display enriched mutations in 4 genes (Mutants) and the other strains isolated from Brazil without such mutations (Wild Types). (A) (B) Box plot for the distribution of the number of indels per stain and average sequencing depth between 47 Mutants and a total of 72 Wild Types. (C) (D) Box plot for the distribution of the number of indels per stain and average sequencing depth between 47 Mutants and randomly selected 47 Wild Types. The p-values of group-wise comparison under Welch's t test are indicated on the top.
